# PyCreas: a tool for quantification of localization and distribution of endocrine cell types in the islets of Langerhans

**DOI:** 10.3389/fendo.2023.1250023

**Published:** 2023-09-12

**Authors:** Melissa Asuaje Pfeifer, Hans Langehein, Katharina Grupe, Steffi Müller, Joana Seyda, Moritz Liebmann, Ingo Rustenbeck, Stephan Scherneck

**Affiliations:** Institute of Pharmacology, Toxicology and Clinical Pharmacy, Technische Universität Braunschweig, Braunschweig, Germany

**Keywords:** prediabetes, gestational diabetes, pancreas, alpha cell, beta cell, delta cell, islet architecture, image analysis

## Abstract

Manifest diabetes, but also conditions of increased insulin resistance such as pregnancy or obesity can lead to islet architecture remodeling. The contributing mechanisms are as poorly understood as the consequences of altered cell arrangement. For the quantification of the different cell types but also the frequency of different cell-cell contacts within the islets, different approaches exist. However, few methods are available to characterize islet cell distribution in a statistically valid manner. Here we describe PyCreas, an open-source tool written in Python that allows semi-automated analysis of islet cell distribution based on images of pancreatic sections stained by immunohistochemistry or immunofluorescence. To ensure that the PyCreas tool is suitable for quantitative analysis of cell distribution in the islets at different metabolic states, we studied the localization and distribution of alpha, beta, and delta cells during gestation and prediabetes. We compared the islet cell distribution of pancreatic islets from metabolically healthy NMRI mice with that of New Zealand obese (NZO) mice, which exhibit impaired glucose tolerance (IGT) both preconceptionally and during gestation, and from C57BL/6 N (B6) mice, which acquire this IGT only during gestation. Since substrain(s) of the NZO mice are known to show a variant in the *Abcc8* gene, we additionally examined preconceptional SUR1 knock-out (SUR1-KO) mice. PyCreas provided quantitative evidence that alterations in the *Abcc8* gene are associated with an altered distribution pattern of islet cells. Moreover, our data indicate that this cannot be a consequence of prolonged hyperglycemia, as islet architecture is already altered in the prediabetic state. Furthermore, the quantitative analysis suggests that states of transient IGT, such as during common gestational diabetes mellitus (GDM), are not associated with changes in islet architecture as observed during long-term IGT. PyCreas provides the ability to systematically analyze the localization and distribution of islet cells at different stages of metabolic disease to better understand the underlying pathophysiology.

## 1 Introduction

Islets of Langerhans have a complex architecture composed of different endocrine cell types that differ in species such as humans and rodents. Rodent islets are characterized by a well-defined structure consisting of a central core of beta cells surrounded by alpha and delta cells in a mantle-like pattern (1, 2). Endocrine cells in islets of human embryos exhibit a distribution pattern similar to that of adult mice (3, 4). In contrast, adult human alpha and delta cells show a diffuse distribution throughout the islet (3). Compared to non-diabetic individuals, the islet architecture of patients with type 2 diabetes is characterized by a decrease in beta cells, amyloid deposition, and an increase in alpha cells localized in the islet center (3, 5–7). A similar central distribution pattern was observed for alpha and delta cells in diabetic and obese db/db mice and for alpha cells in SUR1 knock-out (SUR1-KO) mice, which show mild impaired glucose intolerance (IGT). This alpha cell distribution pattern was also observed in male New Zealand obese (NZO) mice, which represent an established model of type 2 diabetes (8–11). Furthermore, in female NZO mice exhibiting IGT but no manifest diabetes, a distribution of alpha cells near the islet center was observed both before and during gestation (12). During gestation, changes in islet architecture have been observed in CD-1 mice, which exhibit more alpha cells in the islet center, whereas C57BL/6 mice show similar islet architecture during gestation as before gestation. However, it has been observed that during gestation some alpha cells in C57BL/6 mice are localized in the islet center. Increased insulin demand is thought to be responsible for the changes in islet architecture during pregnancy (3, 6, 8).

The mechanisms underlying altered islet architecture include apoptosis, proliferation, dedifferentiation and transdifferentiation. However, these mechanisms are not well understood, nor are the consequences of altered endocrine cell distribution. Furthermore, it has been suggested that the migration of peripheral cells into the islet center may be responsible for altered islet architecture (3, 13, 14). The changes described in the islet architecture are mainly based on qualitative observations. To better understand the underlying mechanisms, it is crucial to describe the differences between metabolic states in a statistically valid manner. Various methods for quantifying the different endocrine cell types in the islets exist (2, 15–20). Moreover, different approaches to determine the frequency of different cell-cell contacts or distances between cells within the islets and between cells and islets are known (16, 21–23). To date, however, only a limited number of methods for statistically valid characterization of the islet cell distribution are available. The published applications for this purpose are often not freely available and limited to a method for evaluating either immunohistochemical staining or immunofluorescence staining. Furthermore, the exact calculations used to determine the localization of the cells are often not made available in detail (24–26).

In this study, we present PyCreas, an open-source tool written in Python for semi-automated analysis of islet cell distribution, capable to analyze images of both immunohistochemistry and immunofluorescence stained pancreatic sections in a statistically valid manner. To validate the glucagon and somatostatin areas within the islets detected by PyCreas, we compared the results with previously published data obtained using the NIS elements AR 5 (Nikon) software (12). To demonstrate the suitability of the PyCreas tool for quantifying cell distribution in the islets at different metabolic states, the localization and distribution of alpha, beta and delta cells was analyzed during gestation and prediabetes. For this purpose, we compared the islet cell distribution of metabolically healthy NMRI mice with that of NZO mice, which exhibit IGT both before and during gestation and thus serve as a model for a gestational diabetes mellitus (GDM) subtype with prediabetes (27, 28). The comparison also included C57BL/6 N (B6) mice that acquire IGT only during gestation and thus represent a common GDM subtype (29). Since it is known that substrain(s) of NZO mice have a variant in the *Abcc8* gene, we also examined alpha and delta cell distribution of preconceptional SUR1-KO mice (30).

## 2 Materials and methods

### 2.1 Animals

All procedures were performed under permits from the ethics committee of the Lower Saxony State Office for Consumer Protection and Food Safety (Oldenburg, Germany; ethics approval number: 33.19-42502-04-17/2462; internal IDs (08.01) TSB TU BS, (05.15) TSB TU BS, and (05.19) TSB TU BS. Female SUR1-KO (Abcc8^tm1Jbry^), NZO (NZO/HIBomDife), B6 (C57BL/6NCrl), and NMRI (NMRI/RjHan) mice were used for this study. The NMRI outbred strain was chosen as a metabolically healthy control due to its normal physiological adaptation to gestation and known robust beta cell physiology (12, 31). Mice were housed in an air-conditioned room at 21 ± 1°C with a lighting period comprised of a 12:12 h light–dark cycle (lights on at 06:30 am). Animals had ad libitum access to water and food (1328 P, Altromin, Lage, Germany) with a content of 11% fat, 24% protein, and 65% carbohydrates with total metabolizable energy of 13.5 kJ/g. Female NZO, B6 and NMRI mice were mated overnight at the age of approximately 7-10 weeks. Gestation was confirmed by the presence of vaginal plugs the following morning. This day was denoted as 0.5 days *post coitum*. Mice were studied at d14.5 of gestation at the age of about 9-12 weeks. The preconceptionally examined mice were the same age as the pregnant ones. SUR1-KO mice were generously provided by Lydia Aguilar-Bryan (9).

### 2.2 Immunofluorescence and immunohistochemical staining of pancreatic sections

After collection, pancreatic tissues were formalin fixed and paraffin-embedded according to standard procedures (32). Thereafter, representative sections of 4 µm and serial sections of 4 μm at sampling intervals of 150 µm were prepared and rehydrated. Immunofluorescence staining was carried out using mouse monoclonal anti-insulin antibody (1:50,000; Sigma-Aldrich, Steinheim, Germany), rabbit polyclonal anti-glucagon antibody (1:100; Cell Marque, Rocklin, USA), and rabbit polyclonal anti-somatostatin antibody (1:1,000; Abcam, Cambridge, UK). Primary antibodies were detected with fluorophore-labelled secondary antibodies Rhodamine Red-X goat anti-mouse (1:200 (insulin); Jackson ImmunoResearch, West Grove, PA, USA) and Alexa Fluor488 goat anti-rabbit (1:500 (glucagon), 1:200 (somatostatin); Jackson ImmunoResearch, West Grove, PA, USA). Nuclei were visualized with DAPI (1:1,000; KPL, Gaithersburg, MD, USA). Imaging was performed with an inverted Eclipse Ti2-E microscope (Nikon, Düsseldorf, Germany) equipped with a CSU W1 spinning disk unit (Yokogawa, Tokyo, Japan) and a sCMOS camera (Prime BSI, Teledyne Photometrics, Tucson, AZ, USA) controlled by VisiView^®^ Premier software (Visitron Systems, Munich, Germany). Immunohistochemical staining was carried out using rabbit polyclonal anti-glucagon antibody (1:200; Cell Marque, Rocklin, USA) and rabbit polyclonal anti-somatostatin antibody (1:2,000; Abcam, Cambridge, UK). Primary antibodies were detected with the secondary antibody Histofine Simple Stain Mouse MAX PO anti-rabbit (Nichirei Biosciences, Tokia, Japan) which was visualized with diaminobenzidine (Dako, Hamburg, Germany) according to the manufacturer’s instructions. Thereafter, nuclei were stained with Mayer’s hematoxylin (Carl Roth, Karlsruhe, Germany). Imaging was performed using an upright microscope Eclipse Ni-E (Nikon, Düsseldorf, Germany) equipped with a DS-Fi3 Color Camera (Nikon, Düsseldorf, Germany) and analysis software NIS elements AR 5 (Nikon, Düsseldorf, Germany). Quantitative analysis of alpha, beta and delta cell distribution with PyCreas was performed on three sectional planes per animal. About 100 islets per strain and condition (n = 4-6 animals per group) of 100 randomly selected images received a manually defined islet border and were analyzed with PyCreas to determine alpha and delta cell distribution. For determination of beta cell distribution, all islets were considered (n = 6 animals per group; 170-244 islets). Islets that showed no staining were excluded from the analysis to avoid potential bias. This is due to the fact that zero staining leads to an incorrect calculation of the cell distribution. Moreover, to avoid a possible bias, islets that could not be clearly distinguished from exocrine tissue due to the acquisition conditions were excluded from the analysis. Quantitative analysis of alpha and delta cell distribution was performed based on immunohistochemical staining and quantitative analysis of beta cell distribution was performed based on immunofluorescence staining. The images of immunohistochemical staining for glucagon and somatostatin on pancreatic sections of NMRI and NZO mice used for quantitative analysis of alpha and delta cell distribution have already been used in a previous publication to determine glucagon and somatostatin areas within the islets (12).

### 2.3 Development and installation of PyCreas software

PyCreas is an open-source software written in the Python (Python 3.8) programming language. The software is based on NumPy, matplotlib, and OpenCV which are part of the core libraries for scientific computing in Python (33–35). PyCreas is freely available from a repository at https://gitlab.com/scherneckgroup/pycreas and compatible with UNIX/Linux, macOS, and Windows. Image analysis can be performed with common image file formats (such as TIFF, JPG and PNG). Instructions for installing the software and using the graphical user interface (GUI) are provided in the repository’s README and instructions file. Moreover, sample images for image analysis are available in the repository. The first release (version 1.0.0) of PyCreas is described and used for the results presented.

### 2.4 Image analysis with PyCreas

#### 2.4.1 Workflow overview

**Figure 1:**
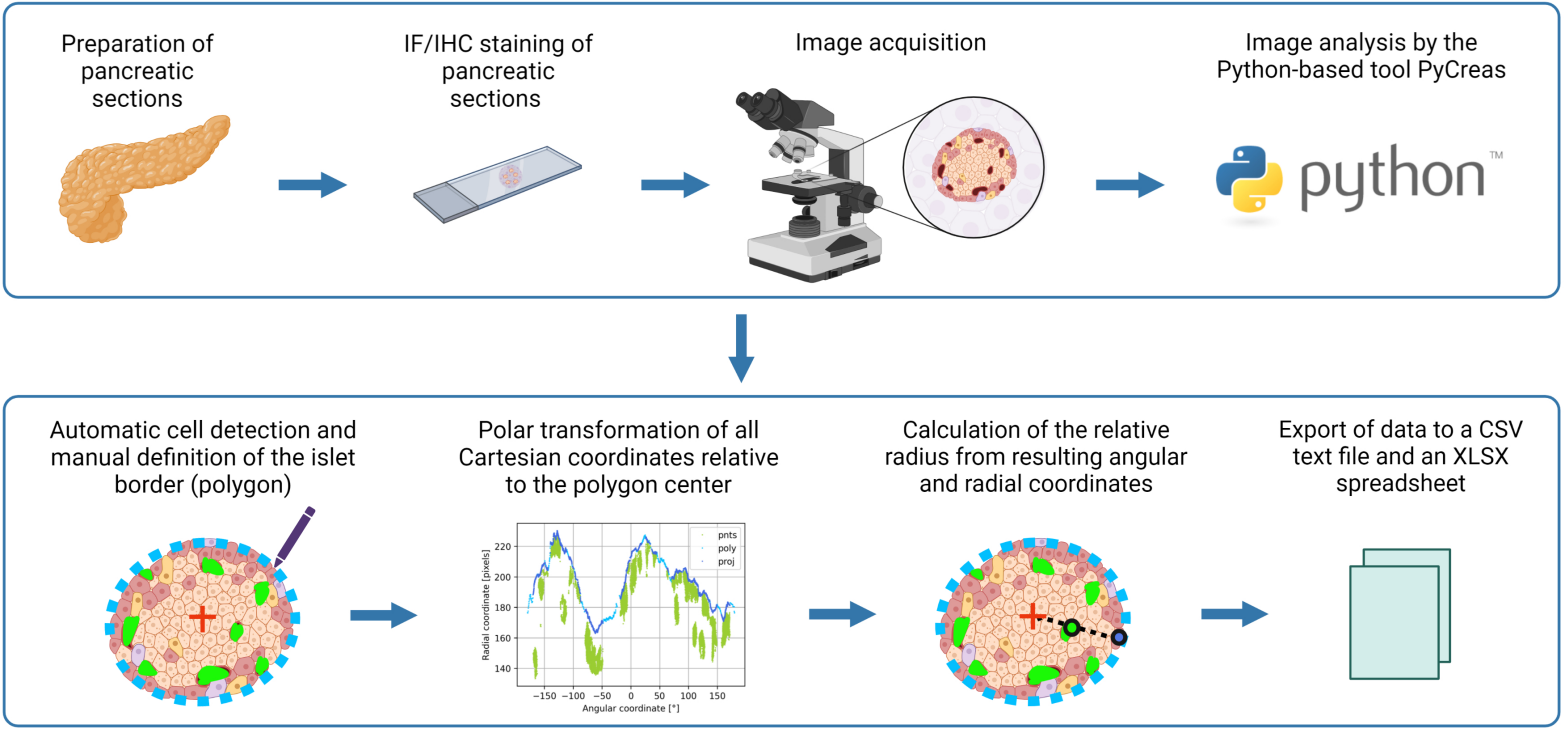
Graphical illustration of the image analysis workflow using PyCreas. Sections of formalin-fixed paraffin-embedded pancreatic tissues were prepared. Sections of the pancreas were stained by immunofluorescence or immunohistochemistry and images were acquired. Image analysis was performed semi-automatically using the Python-based software PyCreas. This included automatic cell detection and manual definition of the islet border by drawing a polygon. Subsequently, a polar transformation of all Cartesian coordinates relative to the polygon center was performed by the software. The resulting angular and radial coordinates were used to calculate the relative radii in order to estimate the cell distribution. Finally, the data was exported to a CSV text file and an XLSX spreadsheet (graphical illustration was created with BioRender.com).

After preparation of pancreatic sections, immunofluorescence or immunohistochemistry staining and image acquisition, quantitative image analysis of the localization and distribution of islet cells was performed using PyCreas. Before starting the program, the TIFF images to be analyzed were imported into a target folder. PyCreas then searched for images in this target folder. Quantitative image analysis involved several steps performed in three subprograms that were either user-controlled or fully automated: CellDetection (automatic detection of cells in the image), IsletGUI (manual definition of an islet border by drawing a polygon), and RelativeRadius (calculation of the relative radius of points within a polygon). The relative radius describes the cell distribution within the islets as the ratio of length between two equidirectional lines that start in the islet center and are bounded by a hormone-positive pixel and the islet border, respectively. The lesser the calculated relative radii, the closer the cells are localized to the islet center. In islets from mice with a more mantle-core phenotype, the relative glucagon and somatostatin radii are expected to be larger than the relative insulin radii. The results were subsequently saved as a CSV text file and as an XLSX spreadsheet (**Figure 1**). Each subprogram is described in detail in the following sections, using the determination of alpha cell distribution in preconceptional NMRI mice as an example for the analysis of immunohistochemical staining and the determination of beta cell distribution in preconceptional NMRI mice as an example for the analysis of immunofluorescence staining.

##### 2.4.1.1 Analysis of immunohistochemical staining

###### 2.4.1.1.1 Subprogram CellDetection

**Figure 2:**
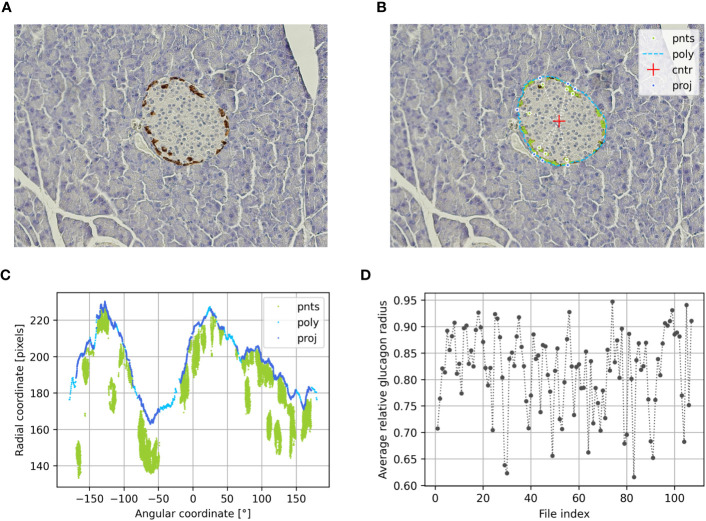
Determination of the alpha cell distribution via the relative glucagon radius of preconceptional NMRI mice using PyCreas. Representative immunohistochemical staining for glucagon (brown) on a pancreatic section of a preconceptional NMRI mouse **(A)** before and **(B)** after automatic cell detection and manual definition of the islet border by drawing a polygon. The light blue line represents the drawn polygon (poly). The detected glucagon-positive pixels are marked in green. They represent points within the polygon (pnts). The red cross represents the polygon center (cntr), while the dark blue points represent the radial projection of each given point onto the polygon sides (proj). **(C)** Transformation of all Cartesian coordinates to polar coordinates including the coordinates of the polygon (poly), the points within the polygon (pnts) and their projections (proj) relative to the polygon center. **(D)** Average relative glucagon radius of each islet of the preconceptional NMRI mice analyzed per image file.

In this subprogram, an image thresholding operation was performed to obtain a mask that isolated the hormone-positive pixels of a range of colors from the rest of the image. The mask provided the location of the detected pixels and was used to automatically detect pixels corresponding to cells within the islet. The lower and upper thresholds were coded in HSV (Hue, Saturation, Value) color space and adjusted to detect an earthy red-brown (**Figures 2A, B**).

###### 2.4.1.1.2 Subprogram IsletGUI

In this subprogram, a GUI was created that allowed to draw a polygon serving as the border of the islet (**Figure 2B**).

###### 2.4.1.1.3 Subprogram RelativeRadius

In this subprogram, the relative radius of each point in a first given set of 2D points was calculated within the polygon defined by a second given set of 2D points.

To compute the relative radii, the center of the polygon was calculated (**Figure 2B**). Thereafter, all Cartesian coordinates were transformed to polar coordinates relative to the polygon center. Thereafter, all Cartesian coordinates were transformed to polar coordinates relative to the polygon center. This was followed by a calculation of the distance to the center and the angle with the horizontal axis. The angular components of the polar coordinates were required for the radial projection of each given point onto the polygon sides by linear interpolation. The radial components of the polar coordinates were required to calculate the relative radius using given points and their projections (**Figure 2C**). Finally, an arithmetic averaging of the relative radii was performed to obtain an islet specific quantification of the cell distribution (**Figure 2D**). Additionally, the relative area was calculated from the ratio of cell pixel area to islet pixel area.

To be able to determine the relative radius, the angular components of the polar coordinates of the polygon must be (weakly) monotonic, that is, only increasing or decreasing. Otherwise the determination of the projection points is ambiguous. Such polygons are called invalid. Invalid polygons are automatically made valid by sorting the polar coordinates with respect to their angles. This changes the original shape of the polygon, which is why the detection of an invalid polygon is reported. Convex polygons are always valid. Concave polygons can, but need not necessarily, be invalid. If the concave regions of an invalid polygon make up a relatively small part, the deviation from the original shape after making the polygon valid is negligible. Due to a modification of the original islet shape, relative radii greater than 100% can occur. Since the relative radius is calculated exclusively for cells within the islet, relative radii greater than 100% are excluded from averaging. To clarify the effect on the average relative radius, the percentage of excluded relative radii is calculated and displayed in the results.

##### 2.4.1.2 Analysis of immunofluorescence staining

###### 2.4.1.2.1 Subprogram CellDetection

**Figure 3:**
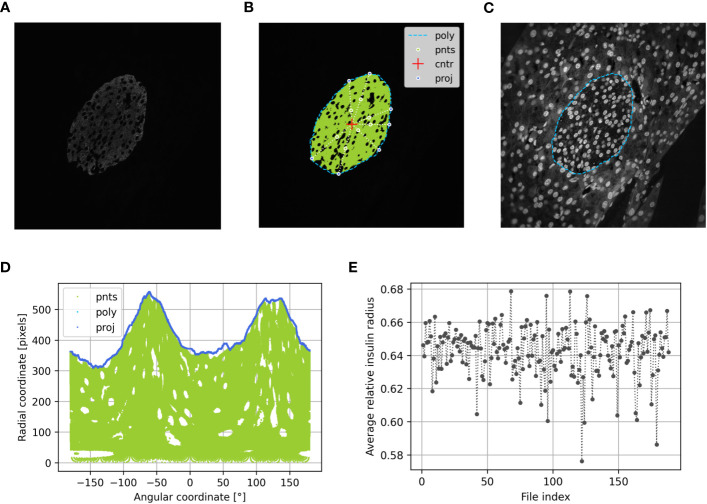
Determination of the beta cell distribution via the relative insulin radius of preconceptional NMRI mice using PyCreas. Representative immunofluorescence staining for insulin (shown by grayscale) on a pancreatic section of a preconceptional NMRI mouse **(A)** before automatic cell detection, **(B)** after automatic cell detection and **(C)** manual definition of the islet border by drawing a polygon using a support image with DAPI staining. The light blue line represents the drawn polygon (poly). Marked in green are the detected insulin-positive pixels which represent points within the polygon (pnts). The red cross represents the polygon center (cntr) and the dark blue points represent the radial projection of each given point onto the polygon sides (proj). **(D)** Transformation of all Cartesian coordinates to polar coordinates including the coordinates of the polygon (poly), the points within the polygon (pnts) and their projections (proj) relative to the polygon center. **(E)** Average relative insulin radius of each islet of the preconceptional NMRI mice analyzed per image file.

As described for analysis of immunohistochemical staining, an image thresholding operation was performed in this subprogram to obtain a mask that isolated the hormone-positive pixels from the rest of the image. Since the evaluation of immunofluorescence staining was performed using grayscale images, light intensity levels were used here as fallback thresholds (**Figures 3A, B**).

###### 2.4.1.2.2 Subprogram IsletGUI

As described for analysis of immunohistochemical staining in this subprogram, a GUI was created that allowed drawing a polygon that served as the border of the islet. In addition to the given main image, support images were added and automatically loaded into the GUI. Images with DAPI staining were used as support images (**Figure 3C**). The support images were used to draw a polygon. The results were processed in the same way as if no support images were provided. Support images were placed in a subfolder named ‘support’ in the same location as the main image. A support image was related to the main image by its filename prefix if the first three characters of the name matched. If the prefix is not unique, the program uses the first match. For convenience, the contrast of the support image was increased. This was done for display only and had no further effect on processing.

###### 2.4.1.2.3 Subprogram RelativeRadius

In this subprogram, the relative radius was calculated as described for analysis of immunohistochemical staining (**Figure 3D**). An arithmetic averaging of the relative radii was performed to obtain an islet specific quantification of the cell distribution (**Figure 3E**). Additionally, the relative area was calculated from the ratio between cell pixel area and islet pixel area.

### 2.5 Statistics

Statistical analysis and graphical presentation were performed using GraphPad Prism 9 (GraphPad, La Jolla, San Diego, CA, USA). Data are presented as means ± *SEM*. To compare differences within one strain over the period of time (e.g., NMRI pc. vs. NMRI d14.5), the Mann-Whitney *U* test was applied. To compare differences between more than two strains, a Kruskal–Wallis *H* test followed by a Dunn’s multiple-comparison test was applied (e.g., NMRI pc. vs. B6 pc. vs. NZO pc. vs. SUR1-KO pc.). Differences were considered significant if *p* < 0.05. *p* values were indicated as * *p* < 0.05, ** *p* < 0.01. Method comparison regarding the determination of glucagon and somatostatin areas within the islets using PyCreas and NIS Elements AR 5 (Nikon) was determined by calculating the Spearman’s rank correlation coefficient (36). This was performed based on previously published data (12).

## 3 Results

### 3.1 Validation of islet hormone quantification with PyCreas

**Figure 4:**
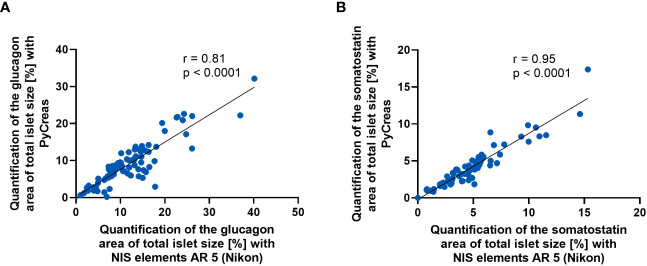
Comparison of glucagon and somatostatin quantification between PyCreas and Nikon software. Spearman’s rank correlation scatter plots comparing **(A)** glucagon and **(B)** somatostatin areas within the islets in preconceptional NMRI mice determined by PyCreas with those determined by NIS elements AR 5 (Nikon). The total number of validated islets was **(A)** *n* = 83 and **(B)** *n* = 68 (blue circles). To ensure that identical islets were validated, only images of single islets were used for validation. The solid black lines represent a linear regression fit to the data.

To validate the glucagon and somatostatin areas within the islets detected by PyCreas, the determination of the glucagon and somatostatin area of total islet size [%] by PyCreas was compared to the determination by the NIS elements AR 5 (Nikon) software. Strong correlation (*r* = 0.81) was observed for the quantification of glucagon areas (**Figure 4A**) and very strong correlation (*r* = 0.95) for the quantification of somatostatin areas (**Figure 4B**). These data confirmed the validity of the semi-automated quantification of glucagon and somatostatin areas within the islets using the PyCreas software.

### 3.2 Analysis of localization and distribution of islet cells before and during gestation

**Figure 5:**
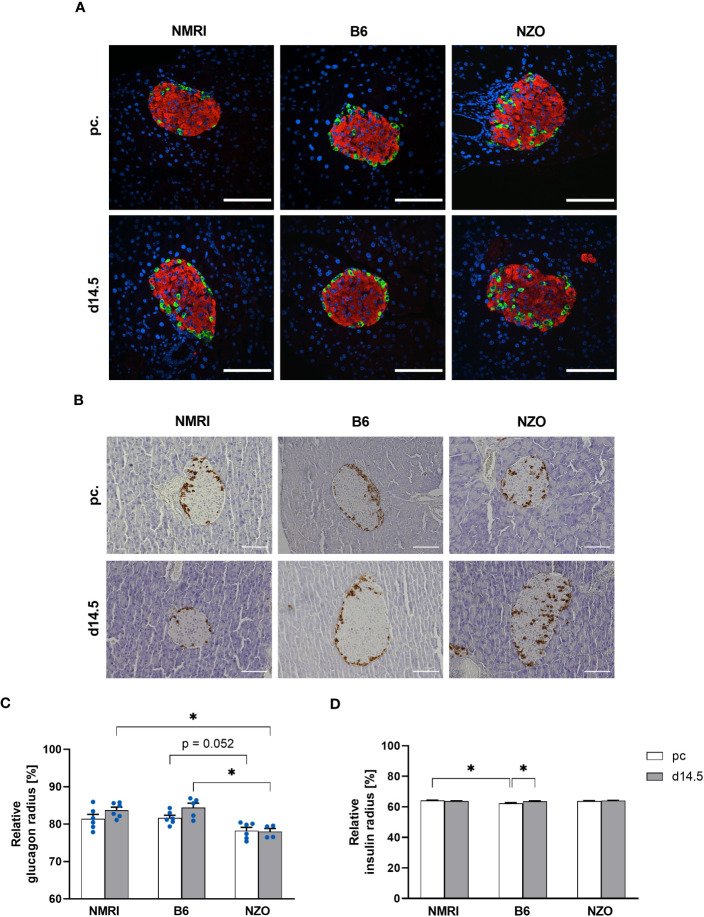
Comparison of the alpha and beta cell distribution before and during gestation. **(A)** Representative double immunofluorescence staining for glucagon (green) and insulin (red) on pancreatic sections of NMRI, B6, and NZO mice at time points preconceptional and day 14.5 of gestation. Nuclei were stained with DAPI (blue). Scale bars, 100 µm. **(B)** Representative immunohistochemical staining for glucagon (brown) on pancreatic sections of NMRI, B6, and NZO mice at time points preconceptional and day 14.5 of gestation. Scale bars, 100 µm. **(C)** Alpha and **(D)** beta cell distribution of NMRI, B6, and NZO mice at time points preconceptional (white bars) and day 14.5 of gestation (gray bars). Quantitative analysis of alpha cell distribution was performed based on immunohistochemical staining and quantitative analysis of beta cell distribution was performed based on immunofluorescence staining. Data are presented as means ± SEM (**(C)** n = 4-6 animals per group; blue circles, **(D)** n = 6 animals per group). *p < 0.05.

**Figure 6:**
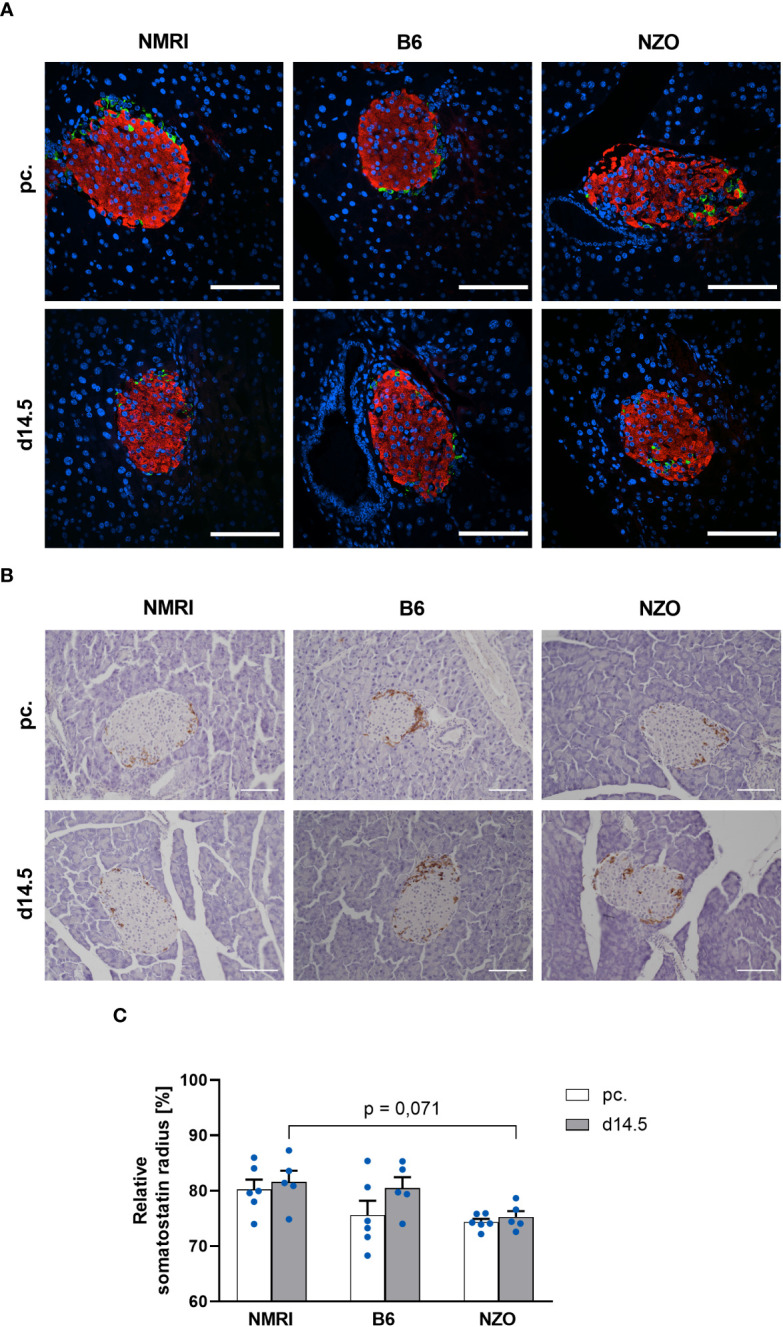
Comparison of the delta cell distribution before and during gestation. **(A)** Representative double immunofluorescence staining for somatostatin (green) and insulin (red) on pancreatic sections of NMRI, B6, and NZO mice at time points preconceptional and day 14.5 of gestation. Nuclei were stained with DAPI (blue). Scale bars, 100 µm. **(B)** Representative immunohistochemical staining for somatostatin (brown) on pancreatic sections of NMRI, B6, and NZO mice at time points preconceptional and day 14.5 of gestation. Scale bars, 100 µm. **(C)** Delta cell distribution of NMRI, B6, and NZO mice at time points preconceptional (white bars) and day 14.5 of gestation (gray bars). Quantitative analysis of delta cell distribution was performed based on immunohistochemical staining. Data are presented as means ± SEM (n = 5-6 animals per group; blue circles).

To verify the suitability of the PyCreas tool for quantitative analysis of islet cell distribution in different metabolic states, the alpha, beta and delta cell distribution of metabolically healthy NMRI mice was compared at the time points preconceptional and day 14.5 of gestation with NZO mice, which exhibit IGT before conception, and with B6 mice, which acquire IGT only during gestation. In the qualitative analysis of the alpha cell distribution using images of immunofluorescence staining, NZO mice showed a random distribution of alpha cells throughout the islet at both time points. In contrast, alpha cells in the B6 and NMRI strains were distributed in the islet periphery (**Figure 5A**). The quantitative analysis of images of immunohistochemical staining (**Figure 5B**) confirmed these observations. Preconceptionally, alpha cells in the NZO mice showed a trend towards a distribution that was closer to the islet center than in the B6 and NMRI strains. This difference was significant during gestation (NZO vs. B6 vs. NMRI, d14.5: 78.01 ± 0.85 vs. 84.42 ± 1.19 vs. 83.75 ± 0.81%; NZO vs. B6: *p* < 0.05, NZO vs. NMRI: *p* < 0.05). No difference was observed between the B6 and NMRI strains at either time point. During gestation, alpha cells in the B6 and NMRI strains showed a trend towards a distribution that was closer to the islet periphery compared to preconceptional, while alpha cell distribution in the NZO mice remained unchanged (**Figure 5C**). In the quantitative analysis of beta cell distribution using images of immunofluorescence staining, B6 mice exhibited a trend towards a distribution near the islet center compared to the NZO mice preconceptionally. This difference was significant when the B6 mice were compared to the NMRI strain, while no difference was observed between the NZO mice and the NMRI strain (NZO vs. B6 vs. NMRI, pc.: 63.81 ± 0.20 vs. 62.36 ± 0.44 vs. 64.21 ± 0.19%; B6 vs. NMRI: *p* < 0.05). At day 14.5 of gestation, no difference in beta cell distribution was observed between the three mouse strains. During gestation, beta cells of the B6 strain were distributed significantly closer to the islet center compared to preconceptional (B6 d14.5 vs. B6 pc.: 63.60 ± 0.32 vs. 62.36 ± 0.44%; *p* < 0.05). In contrast, in the NZO and NMRI mice, no difference was exhibited during gestation compared to the state before conception (**Figure 5D**). The qualitative analysis of delta cell distribution using images of immunofluorescence staining showed a random distribution of δ-cells throughout the islet at both time points in the NZO mice. In contrast, delta cells in the B6 and NMRI strains were distributed in the islet periphery (**Figure 6A**). This was confirmed by the quantitative analysis of the delta cell distribution using images of immunohistochemical staining (**Figure 6B**). At both time points, delta cells in NZO mice showed a trend towards a distribution that was closer to the islet center compared to B6 and NMRI mice. This was also observed in B6 mice compared to NMRI mice at both time points. During gestation, all three strains showed a trend towards a more peripheral distribution of the delta cells compared to the state before conception (**Figure 6C**). NZO mice but not B6 mice exhibited an altered distribution pattern of alpha and delta cells at both time points.

### 3.3 Analysis of localization and distribution of islet cells in SUR1-KO mice

**Figure 7:**
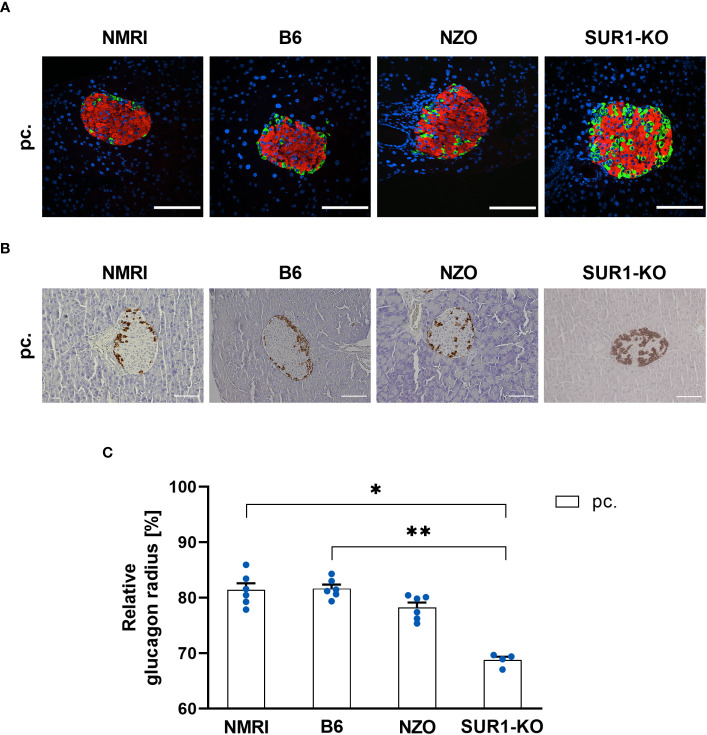
Determination of the alpha cell distribution in SUR1-KO mice. **(A)** Representative double immunofluorescence staining for glucagon (green) and insulin (red) on pancreatic sections of preconceptional NMRI, B6, NZO, and SUR1-KO mice. Nuclei were stained with DAPI (blue). Scale bars, 100 µm. The apparently altered number of alpha cells in the SUR1-KO mice was not the subject of the present work. However, it was previously published as significantly increased glucagon content by Früh et al. (10). **(B)** Representative immunohistochemical staining for glucagon (brown) on pancreatic sections of NMRI, B6, NZO, and SUR1-KO mice at time point preconceptional. Scale bars, 100 µm. **(C)** Alpha cell distribution of preconceptional NMRI, B6, NZO, and SUR1-KO. Quantitative analysis of alpha cell distribution was performed based on immunohistochemical staining. Data are presented as means ± SEM (n = 4-6 animals per group; blue circles). *p < 0.05, **p < 0.01.

**Figure 8:**
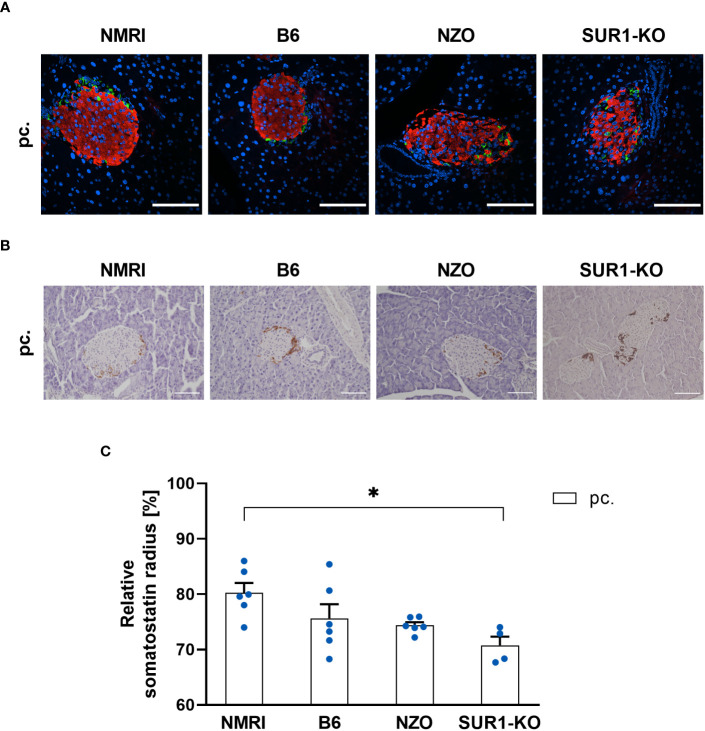
Determination of the delta cell distribution in SUR1-KO mice. **(A)** Representative double immunofluorescence staining for somatostatin (green) and insulin (red) on pancreatic sections of preconceptional NMRI, B6, NZO, and SUR1-KO mice. Nuclei were stained with DAPI (blue). Scale bars, 100 µm. **(B)** Representative immunohistochemical staining for somatostatin (brown) on pancreatic sections of NMRI, B6, NZO, and SUR1-KO mice at time point preconceptional. Scale bars, 100 µm. **(C)** Delta cell distribution of preconceptional NMRI, B6, NZO, and SUR1-KO mice. Quantitative analysis of delta cell distribution was performed based on immunohistochemical staining. Data are presented as means ± SEM (n = 4-6 animals per group; blue circles). *p < 0.05.

To investigate possible associations between alterations in the *Abcc8* gene of the NZO mice and islet architecture, the distribution of alpha and delta cells was analyzed in preconceptional SUR1-KO mice. The qualitative analysis of alpha cell distribution using images of immunofluorescence staining showed a random distribution of alpha cells throughout the islet in SUR1-KO mice. Thereby, the alpha cells were localized closer to the islet center compared to the NZO mice (**Figure 7A**). The quantitative analysis of images of immunohistochemical staining (**Figure 7B**) confirmed these observations. The alpha cells of the SUR1-KO showed a trend towards a distribution that was closer to the islet center compared to NZO mice. This difference was significant when the SUR1-KO mice were compared to the B6 and NMRI strains (SUR1-KO vs. NZO vs. B6 vs. NMRI, pc.: 68.79 ± 0.59 vs. 78.25 ± 0.89 vs. 81.68 ± 0.71 vs. 81.44 vs. 1.19%; SUR1-KO vs. B6: *p* < 0.01, SUR1-KO vs. NMRI: *p* < 0.05) (**Figure 7C**). The qualitative analysis of delta cell distribution using images of immunofluorescence staining showed that delta cells in SUR1-KO mice were localized closer to the islet center, comparable to the distribution pattern in NZO mice (**Figure 8A**). In the quantitative analysis, using images of immunohistochemical staining (**Figure 8B**), delta cells of the SUR1-KO mice exhibited a trend towards a distribution that was closer to the islet center compared to the NZO and B6 strain. This difference was significant when the SUR1-KO mice were compared to the NMRI strain (SUR1-KO vs. NZO vs. B6 vs. NMRI, pc.: 70.73 ± 1.59 vs. 74.36 ± 0.57 vs. 75.63 ± 2.56 vs. 80.26 ± 1.75%; SUR1-KO vs. NMRI: *p* < 0.05) (**Figure 8C**). The SUR1-KO mice showed an alteration in the distribution pattern of the alpha and beta cells, which was more pronounced than in the NZO mice.

## 4 Discussion

This study introduces PyCreas, a novel tool for semi-automated quantitative analysis of islet cell distribution based on immunohistochemical and immunofluorescence images of pancreatic sections. In order to validate the glucagon and somatostatin areas within the islets detected by PyCreas, we compared our results with data generated by the NIS elements AR 5 (Nikon) software. Furthermore, we demonstrated the suitability of the PyCreas tool to quantify islet architecture in different metabolic states. For this purpose, alpha, beta and delta cell distribution of metabolically healthy NMRI mice was compared at the time points preconceptional and day 14.5 of gestation with NZO mice, which exhibit IGT before conception, and with B6 mice, which acquire IGT only during gestation. Furthermore, to investigate possible associations between alterations in the *Abcc8* gene of NZO mice and islet architecture, the distribution of alpha and delta cells was analyzed in preconceptional SUR1-KO mice.

With the development of PyCreas, we have created a new open-source tool for fast and accurate quantitative analysis of islet cell distribution, regardless of whether the analysis is based on immunofluorescence or immunohistochemistry. The developed algorithm consists of three subprograms that provide image thresholding to distinguish the relevant pixels from the background, a user-friendly GUI to draw the islet border for generating a polygon, and the calculation of the relative radius to analyze precisely where a hormone-positive pixel is localized within the polygon. Validation of the quantification of glucagon and somatostatin areas within the islets showed that the results obtained by PyCreas highly correlated with results from NIS elements AR 5 (Nikon) software. This indicates that PyCreas is able to correctly distinguish the hormone-positive pixel from the background. However, for a comprehensive study of islet architecture, in addition to analysis of islet cell localization and distribution, determination of islet vascularization or innervation and quantification of differences in key extracellular matrix proteins is indicated. Quantification of the localization of these islet components can be performed with PyCreas after appropriate staining and validation.

Using PyCreas, we analyzed the distribution of alpha, beta and delta cells in different metabolic states. The quantitative image analysis showed that preconceptionally, the beta cells of the B6 strain were distributed closer to the islet center compared to the NMRI strain and the NZO mice. A significant difference was observed compared to the NMRI strain. This may be partly due to a slightly reduced beta cell size in the B6 strain. The alpha cells of the NZO mice were distributed closer to the islet center compared to the B6 and NMRI strains both preconceptionally and during gestation, with this difference being significant during gestation. The obtained results are in agreement with the observation previously described by our group indicating that the alpha cells of NZO mice are diffusely distributed throughout the islet area (12). A trend towards a more central distribution of delta cells was also observed in the NZO mice compared to the B6 and NMRI strains before and during gestation. Since previously published data showed no significant change in insulin content in NZO mice compared to NMRI mice, it is unlikely that the observed differences in cell distribution are due to altered beta cell mass (12). The changes in islet architecture NZO mice may be explained by beta cell dedifferentiation and conversion to alpha and delta cells or transdifferentiation of beta cells into alpha cells, which has been previously described in studies of diabetic mice and humans with type 2 diabetes (37–40). Brereton et al. attributed changes in islet structure to hyperglycemia and excluded activation of K_ATP_ channels as a cause in the mouse model studied, which had a mutation in the Kir6.2 subunit of K_ATP_ channels. The changes in islet structure were prevented by insulin therapy and completely reversed by sulfonylurea administration (40). However, the altered alpha cell distribution observed in the NZO mouse cannot be the result of sustained hyperglycemia, as the islet architecture already shows changes in the prediabetic stage. Since substrain(s) of the NZO mice show a variant in the *Abcc8* gene, and quantitative analysis confirmed that alpha cells and delta cells in islets of SUR1-KO mice are distributed closer to the islet center compared to NZO mice, this suggests a relationship between alterations in the *Abcc8* gene and islet architecture (30). Stancill et al. showed that mice lacking *Abcc8* exhibit beta cell to pancreatic polypeptide (PP) cell transdifferentiation and increased expression of *Aldh1a3*, a marker of dedifferentiating beta cells. Moreover, they observed a reduction in multiple cell adhesion molecules. This leads to the hypothesis that altered islet architecture in NZO mice is also associated with loss of beta cell identity or impaired cell adhesion mentioned by the authors, and provides an approach for further investigation (41). Furthermore, this suggests that the remodeling of islet architecture is a consequence of altered K_ATP_ channel function. Functional K_ATP_ channels may therefore be essential for the physiological arrangement of cells and their connection to the vasculature, and alterations in their function could lead to pathological islet architecture remodeling. A possible link between alterations in the *Abcc8* gene and islet architecture is further supported by previous studies from our group showing increased glucagon areas in the islets of NZO mice before and during gestation, which is consistent with data from Früh et al. who observed increased glucagon content in the islets of SUR1-KO mice (10, 12). Andrikopoulos et al. showed that substrain(s) of the NZO strain exhibit reduced *Abcc8* expression levels, however, to date it is not known whether the genetic defect in *Abcc8* in NZO mice leads to a gain or loss-of-function of the K_ATP_ channels (30). In beta cells from transgenic mice expressing a dominant negative form of the Kir6.2 subunit of K_ATP_ channels and in Kir6.2-KO mice, the distribution of alpha cells is comparable to that in SUR1-KO mice (42, 43). This suggests that K_ATP_ channel dysfunction is independent of the subunit involved and that complete loss of K_ATP_ channels is associated with changes in islet architecture. Since the differences in the distribution of alpha and delta cells were more pronounced in SUR1-KO mice compared to NZO mice, this indicates that complete loss of K_ATP_ channels is associated with more pronounced changes in islet architecture than K_ATP_ channel dysfunction. Since B6 mice that acquire IGT only during gestation do not show changes in islet cell distribution, as is the case in NZO mice and SUR1-KO mice with preconceptional IGT, this implies that only long-term IGT or manifest diabetes as described in the literature are associated with altered islet architecture (3, 5–7).

During gestation, beta cells of the B6 strain were localized significantly closer to the islet periphery, which may indicate an increase in beta cell size. However, no difference in beta cell distribution was observed in the other strains. A trend towards a distribution of alpha and delta cells closer to the islet periphery was observed during gestation, although the distribution of alpha cells remained unchanged in NZO mice. The small changes in beta cell distribution during gestation are consistent with the small changes in beta cell mass previously described by our group in NZO and NMRI mice. Here we showed that both strains exhibit a slight increase in insulin content during gestation, which is associated with an increase in islet size and a decrease in glucagon areas within the islets. This was also accompanied by a decrease in somatostatin areas in the islets of NMRI mice and a slight increase in NZO mice (12). The small changes in beta cell distribution and mass support the suggestion that, in the absence of changes in beta cell mass, enhanced beta cell functionality is responsible for the increase in insulin secretion to meet the higher insulin demand during gestation (44). Moreover, our results are supported by a study showing that the mantle-core structure of islets in mice remains unchanged despite an increase in alpha and beta cell mass induced by insulin and glucagon receptor inhibition. Therefore, it is assumed that there is no change in islet architecture even with a compensatory increase in islet mass (45, 46). In studies, some alpha cells have been found to be closer to the islet center during gestation in mice (3, 6, 8). However, this is in contrast to the trend observed in this work, where alpha cells from B6 and NMRI mice were found to localize closer to the islet periphery during gestation. The differences in islet cell distribution in pregnant mice may be a consequence of different metabolic states during gestation or gene variants, such as a variant in the *Abcc8* gene as described here. However, it is also conceivable that these differences are due to the fact that the assessment of cell distribution in these studies was performed only by qualitative observation of individual islets, highlighting the importance of systematic statistical analysis of islet cell distribution.

In conclusion, using Pycreas, we have shown that alterations in the *Abcc8* gene are associated with an altered islet distribution pattern. However, this cannot be a consequence of prolonged hyperglycemia because islet architecture is already altered in the prediabetic state. Furthermore, our data suggest that common GDM with acquired IGT is not associated with changes in islet architecture as observed during long-term IGT. Thus, PyCreas allows accurate quantitative analysis of the localization and distribution of islet cells in different metabolic states in order to better understand the underlying pathophysiology.

## Data availability statement

The raw data supporting the conclusions of this article will be made available by the authors, without undue reservation.

## Ethics statement

The animal study was approved by the Ethics Committee of the Lower Saxony State Office for Consumer Protection and Food Safety (Oldenburg, Germany; ethics approval number: 33.19‐42502‐04‐17/2462; internal IDs (08.01) TSB TU BS, (05.15) TSB TU BS, and (05.19) TSB TU BS) and the animal welfare representative of the Technische Universität Braunschweig. The study was conducted in accordance with the local legislation and institutional requirements.

## Author contributions

MAP, HL, and SS conceived and designed research. MAP, HL, KG, SM, JS, and ML performed experiments, analyzed data, and prepared figures. MAP, HL, and SS interpreted results of conducted experiments. MAP drafted the manuscript. MAP, HL, KG, JS, IR, and SS reviewed, edited, and wrote the final version of the manuscript. All authors contributed to the article and approved the submitted version.
